# Clinical and biochemical predictors and predictive model of primary aldosteronism

**DOI:** 10.1371/journal.pone.0272049

**Published:** 2022-08-05

**Authors:** Worapaka Manosroi, Natthanan Tacharearnmuang, Pichitchai Atthakomol

**Affiliations:** 1 Endocrine and Metabolism Unit, Internal Medicine Department, Faculty of Medicine, Chiang Mai University, Muang Chiang Mai, Chiang Mai, Thailand; 2 Internal Medicine Department, Lee Hospital, Lamphun, Thailand; 3 Orthopedics Department, Faculty of Medicine, Chiang Mai University, Muang Chiang Mai, Chiang Mai, Thailand; Medical University of Bahrain, BAHRAIN

## Abstract

**Background:**

Primary aldosteronism (PA) is the most common cause of secondary hypertension. The diagnosis of PA currently requires multiple complicated measures. The aims of this study were to identify easy-to-obtain clinical and biochemical predictors, and to create predictive model to facilitate the identification of a patient at high risk of having PA.

**Materials and methods:**

This 2-year retrospective cohort study was conducted at a tertiary care medical center. A total of 305 patients who had been tested for plasma aldosterone concentration (PAC) and plasma renin activity (PRA) were identified. Patients with incomplete results of PAC and PRA and those who had an established diagnosis of Cushing’s syndrome or pheochromocytoma were excluded. Logistic regression analysis was used to identify significant predictors and to create predictive model of PA.

**Results:**

PA was diagnosed in 128 of the patients (41.96%). Significant predictive factors for PA were age >60 years (OR 2.12, p = 0.045), female (OR 1.65, p<0.001), smoking (OR 2.79, p<0.001), coronary artery disease (OR 2.29, p<0.001), obstructive sleep apnea (OR 1.50, p = 0.017), systolic blood pressure >160 mmHg (OR 1.15, P<0.001), serum potassium <3 mEq/L (OR 3.72, p = 0.030), fasting blood glucose >126 mg/dL (OR 0.48, p = 0.001) and estimated glomerular filtration rate (eGFR) <60 mL/min/1.73m^2^ (OR 1.79, p = 0.001). Predictive model was created with a total score ranged from 0 to 42. A score above 7.5 indicated a higher probability of having PA with a sensitivity of 72% and a specificity of 70%. The diagnostic performance of the predictive model based on area under the curve was 71%.

**Conclusions:**

The clinical and biochemical predictive factors including predictive model identified in this study can be employed as an additional tool to help identify patients at risk of having PA and could help reduce the number of screening and confirmation tests required for PA.

## Introduction

The most common cause of secondary hypertension is primary aldosteronism (PA) which accounts for 5 to 10% of hypertensive patients and 20% of resistant hypertensive patients [1]. Diagnosis of this condition is beneficial for patients as this is one of the treatable and curable forms of hypertension [2, 3]. If left undiagnosed or inappropriately treated, PA can result in an increased risk of cardiovascular disease, stroke and other target organ damage compared to essential hypertension [4].

Recent screening guidelines for PA identified various indications which indicate an increased likelihood of of PA [5, 6]. The diagnostic procedure for PA involves multiple steps including screening, confirmation, subtype classification and a localization process [5]. Plasma aldosterone concentration (PAC), plasma renin activity (PRA) or direct renin concentration (DRC) are the preferred tests to screen for PA. During the screening process, patients are compelled to discontinue multiple anti-hypertensive medications and close monitoring of blood pressure is required, a complicated procedure which requires professional care. The facilities for laboratory diagnosis may not be available in some institutions. Moreover, in many institutions, there are insufficient trained health-care professionals and endocrinologists to conduct and interpret the tests, interventionists to perform adrenal venous sampling (AVS), and surgeons to perform laparoscopic adrenalectomy. Some patients may need to be referred to another hospital where the resources are accessible, a money- and time-consuming process. Additionally, many of the patients referred to another institution are found to have normal PA screening results.

If easy-to-obtain clinical and biochemical predictive factors for PA could be identified, it could facilitate the diagnosis of PA and also reduce the number of tests for PA screening. To the best of our knowledge, only one study has reported predictive factors and constructed predictive scores for diagnosing PA [7]. Reported factors in that study include age, body mass index (BMI), diabetes status, number of antihypertensive agents taken as well as serum sodium and potassium levels. However, that study used a case detection approach based on the Endocrine Society Clinical Practice Guideline of 2008 which did not include screening of patients with obstructive sleep apnea and hypertension. Those patients are now recommended to be screened for PA in the new 2016 guideline [5, 7, 8].

This study aimed to identify clinical and biochemical predictive factors and to create the predictive model which could facilitate the prediction of PA.

## Materials and methods

A 2-year retrospective cohort study was conducted using data acquired from electronic medical records at Maharaj Nakorn Chiang Mai Hospital, a tertiary care hospital in northern Thailand, during December 2017 to December 2019. The inclusion criteria were patient age over 18 years who had been identified for PA screening according to the 2016 Endocrine Society Clinical Practice Guideline, by using plasma aldosterone concentration (PAC) and plasma renin activity (PRA), to calculate the aldosterone-renin ratio (ARR) [8]. Indications of PA screening according to that guideline include moderate to severe hypertension, resistant hypertension, hypertension with spontaneous or diuretic-induced hypokalemia, hypertension with adrenal incidentaloma, hypertension and a family history of early-onset hypertension or cerebrovascular disease beginning at age <40 years and hypertension with obstructive sleep apnea (OSA). In our institution, patients in the young population (age <40 years) with hypertension were also routinely screened for PA. Exclusion criteria for this study were patients with incomplete results of PAC and PRA and those who had an established diagnosis of Cushing’s syndrome or pheochromocytoma. Demographic data, indications for screening, clinical presentations and laboratory investigation results were retrieved from hospital medical records and reviewed by the investigators. The study was approved by Chiang Mai University Research Ethic Committee (CMUREC).

### Diagnostic criteria

Our institution adopted diagnostic criterion of primary aldosteronism from the 2016 Endocrine Society Clinical Practice Guideline. In brief, PAC and PRA are measured with appropriate patient preparation. Positive screening results for PA include the following: (1) ARR >20 (ng/dL)/(ng/mL/hr), (2) PAC levels of >15 ng/dL and (c) PRA <1.0 ng/mL/hr. Those who meet all the screening criteria proceed to confirmation tests unless the patients has either: (1) spontaneous hypokalemia, undetectable PRA and PAC >20 ng/dL, or (2) ARR >100 (ng/dL)/(ng/mL/hr) [8, 9]. Those who have negative screening results and confirmation tests are diagnosed as essential hypertension.

### Aldosterone-renin ratio (ARR) measurement

Standard procedures for ARR measurement are as follows. Before PAC and PRA are collected, the patient should have a liberal sodium intake and be in a normokalemic state. Antihypertensive medications which interfere with the interpretation of ARR should be discontinued 2–4 weeks before blood is drawn. To maintain optimal blood pressure, antihypertensive medications with less effect on ARR are begun including extended-release verapamil, hydralazine and doxazosin. Blood collection is conducted midmorning after the patients have been upright for at least 2 hours and then seated for 5–15 minutes. The blood is collected in EDTA tubes. PAC and PRA are performed using the enzyme-linked immunoassay method (ELISA) (DIASource Immunoassays^®^, Nivelles, Belgium) [8]. The intra-assay variability for PAC was 4.7–8.5% and for PRA was 5.3–7.1%.

### Confirmatory tests

The saline infusion test (SIT) is the confirmation measure employed in our institution. Four liters of 0.9% normal saline are infused intravenously over 4 hours and PAC is collected before and after the infusion. If post-infusion PAC is >10 ng/dL, PA is confirmed. If post-infusion PAC is <5 ng/mL, PA can be ruled out. Repeated SIT are conducted in patients with an equivocal post-infusion PAC number between 5–10 ng/dL. Computerized tomography and adrenal venous sampling are performed to differentiate PA subtypes and to make a distinction between unilateral and bilateral adrenal diseases.

### Definitions

In this study, moderate to severe hypertension was defined as sustained elevated blood pressure >160/100 mmHg. Resistant hypertension was defined as blood pressure >140/90 mmHg while on three antihypertensive medications including one diuretic. Hypertension with spontaneous or diuretic-induced hypokalemia was defined as documented serum potassium <3.5 mEq/L regardless of the diuretics used. Hypertension with adrenal incidentaloma was defined as an adrenal tumor discovered in hypertensive patients on imaging performed for any reason unrelated to adrenal diseases. Hypertension in the young population was defined as hypertensive patients whose onset of hypertension occurred before 40 years of age. Hypertension with OSA was diagnosed and confirmed by polysomnography. A history of smoking was defined as smoking regularly during the past 6 months. Clinical presentations of PA documented in the medical records included headache and proximal muscle weakness. The estimated glomerular filtration rate (eGFR) was calculated using the CKD-EPI method.

### Statistical analysis

Statistical analysis was performed using the STATA program. The statistical significance was specified as a p-value <0.05 for two-tailed tests. Data are shown as count and percentage or mean and standard deviation (SD) for categorical and continuous data, respectively. Fisher’s exact test and either the t-test or the Mann-Whitney U test were performed for inferential statistics with categorical and continuous data, respectively. Univariable and multivariable analyses of predictive factors for PA were performed using logistic regression analysis clustered by confirmation test and reported as odds ratios (ORs) with a 95% confidence interval. The predictive factors analyzed in the multivariate analysis model were selected based on p-values <0.1 in univariable analysis or on having clinically significant relationship to PA. Stepwise selection with backward elimination by removing variables with p-value >0.2 was conducted to create the final prediction model. Continuous variables were transformed to categorical variables in the final predictive model based on median values. Item scores were calculated by the transformation of the regression coefficient. The coefficient of each level for each factor was divided by the smallest coefficient of the model and rounded to the nearest 0.5. Item scores were then added together to calculate a total score. The total scores were then divided into 2 groups: groups at a low risk and at a high risk of having PA. The cut-off point for the risk levels was retrieved from the level that yielded the highest sensitivity, specificity. To evaluate the performance of the predictive model, the area under the receiver operating characteristics curve (AuROC) was used. Variables with missing data >5% were subjected to multiple imputation regression analysis.

## Results

### Baseline characteristics of predictive factors associated with primary aldosteronism

Of the 305 patients who were screened for PA, 41.96% (128/305) were diagnosed with PA. Of these, 36.7% (47/128) had an aldosterone-producing adenoma and 63.3% (81/128) had idiopathic bilateral adrenal gland hyperplasia. Males had a non-significantly higher prevalence of PA than females. The overall mean age was 43.14±0.90 years, with the mean age significantly higher in the PA group (47.07±15.79) than in the essential hypertension group (40.30±15.14). A history of smoking was more commonly found in the PA than in the essential hypertension group. Among all patients, the most common indications for PA screening were hypertension in the young (68.52%, 209/305) followed by patients with moderate to severe hypertension (36.39%,111/305). Among the indications for PA screening, hypertension with hypokalemia and OSA were more commonly documented in PA patients than in the essential hypertension group. In those with PA, proximal muscle weakness was more frequently reported than in the essential hypertension group. Duration of hypertension was longer in the PA than in the essential hypertension group. Other clinical characteristics, including BMI, co-morbidities, blood pressure level, number of antihypertensive agents and type of anti-hypertensive agents revealed no statistically significant difference between the two groups. Baseline laboratory investigations found a statistically significant lower serum potassium level in the PA than in the essential hypertension group. Other laboratory values showed no statistically significant differences between the groups. Baseline characteristics are shown in **Table 1**.

**Table 1:** Baseline characteristics of patients.

Characteristics	Primary aldosteronism	Essential hypertension	*P* value
	(N = 128)	(N = 177)	
**Demographic data**			
Female, N (%)	62 (48.44)	67 (37.85)	0.065
Age in years, mean (±SD)	47.07 (15.79)	40.30 (15.14)	<0.001
Age at onset of hypertension in years, mean (±SD)	38.46 (1.22)	32.66 (0.95)	0.999
BMI, kg/m2, mean (±SD)	26.04 (5.94)	27.81 (6.18)	0.314
Smoking, N (%)	23 (17.96)	18 (10.16)	0.050
**Co-morbidities**			
Coronary artery disease, N (%)	3 (2.34)	1 (0.56)	0.313
Cerebrovascular disease, N (%)	13 (10.16)	13 (7.34)	0.386
Chronic kidney disease, N (%)	8 (6.25)	10 (5.65)	0.826
Diabetes mellitus, N (%)	14 (10.94)	26 (14.69)	0.338
Hyperlipidemia, N (%)	39 (30.47)	55 (31.07)	0.910
**Indications for investigation**			
Hypertension in the young (age <40 years), N (%)	77 (60.16)	132 (74.58)	0.007
Hypertension with hypokalemia, N (%)	58 (45.31)	47 (26.55)	0.001
Hypertension with adrenal incidentaloma, N (%)	21 (16.41)	35 (19.77)	0.453
Moderate/severe hypertension, N (%)	48 (37.50)	63 (35.59)	0.733
Resistant hypertension, N (%)	4 (3.12)	3 (1.69)	0.418
Hypertension with obstructive sleep apnea, N (%)	29 (16.38)	12 (9.38)	0.077
**Clinical manifestations**			
Proximal muscle weakness, N (%)	14 (10.94)	9 (5.08)	0.056
Headache, N (%)	36 (28.13)	52 (29.38)	0.812
No symptoms, N (%)	80 (62.5)	118 (66.67)	0.452
**Hypertension**			
Duration of hypertension (years) median (IQR)	7 (4–11)	6 (3–11)	0.089
Systolic blood pressure (mmHg) mean (±SD)	147.97 (17.21)	147.50 (17.49)	0.880
Diastolic blood pressure (mmHg) mean (±SD)	89.70 (16.66)	89.37 (13.41)	0.097
**Treatment**			
Number of antihypertensive drugs, mean (IQR)	2 (1–3)	2 (1–2)	0.448
< 3 drugs, N (%)	92 (71.88)	136 (76.84)	0.325
≥ 3 drugs, N (%)	36 (28.12)	41 (23.16)	
**Type of antihypertensive drugs**			
ACEI/ARBs, N (%)	51 (39.84)	72 (40.68)	0.883
Alpha-blocker, N (%)	28 (21.88)	35 (19.77)	0.655
Beta-blocker, N (%)	25 (19.53)	28 (15.82)	0.398
Calcium channel blocker, N (%)	107 (83.59)	143 (80.79)	0.530
Thiazide diuretics, N (%)	11 (8.59)	12 (6.78)	0.554
Loop diuretics, N (%)	0	2 (1.13)	0.511
Spironolactone, N (%)	6 (4.69)	3 (1.69)	0.173
Vasodilator, N (%)	25 (19.53)	39 (22.03)	0.596
**Lab investigations**			
Sodium, mmol/L, mean (±SD)	140.23 (3.08)	139.50 (2.68)	0.306
Potassium, mmol/L, mean (±SD)	3.51 (0.56)	3.77 (0.48)	0.064
Bicarbonate, mmol/L, mean (±SD)	25.08 (3.17)	24.33 (2.53)	0.174
Plasma aldosterone concentration, ng/dL, median (IQR)	36.94 (21.58–65.90)	18.00 (11.48–31.12)	<0.001
Plasma renin activity, ng/mL/hr, median (IQR)	0.29 (0.13–0.71)	1.34 (0.61–3.09)	<0.001
Fasting blood glucose, mg/dL, mean (±SD)	104.97 (38.94)	103.26 (25.97)	0.190
Total cholesterol, mg/dL, mean (±SD)	182.12 (44.85)	184.82 (40.87)	0.226
LDL, mg/dL, mean (±SD)	121.13 (39.65)	126.85 (41.03)	0.307
HDL, mg/dL, mean (±SD)	52.91 (16.44)	59.61 (14.98)	0.192
Creatinine, mg/dL, median (IQR)	0.84 (0.71–1.08)	0.92 (0.74–1.06)	0.405
eGFR, mL/min/1.73m2, mean (±SD)	91.77 (24.20)	95.66 (24.47)	0.197

### Univariate and multivariate logistic regression analysis of predictive factors associated with primary aldosteronism

The predictive factors analyzed in the multivariate analysis model were selected based on p-values <0.1 in univariable analysis or on having clinically significant relationship to PA. Hypertension in the young as an indication for screening and proximal muscle weakness were not incorporated in the final multivariate model due to redundancy with age and serum potassium level, respectively. Continuous predictive variables were transformed into categorical variables for ease of interpretation. After stepwise backward elimination of predictive factors for PA, nine clinical and laboratory predictors were identified in the final predictive model. Those factors consisted of age > 60 years (OR 2.12, p = 0.045), female (OR 1.65, p<0.001), smoking (OR 2.79, p<0.001), coronary artery disease (OR 2.29, p<0.001), OSA (OR 1.50, p = 0.017), systolic blood pressure > 160 mmHg (OR 1.15, P<0.001), serum potassium < 3 mEq/L (OR 3.72, p = 0.030), fasting blood glucose > 126 mg/dL (OR 0.48, p = 0.001) and eGFR < 60 mL/min/1.73m^2^ (OR 1.79, p = 0.001). Data are shown in **Table 2**.

**Table 2:** Univariable and multivariable logistic regression analyses of predictive factors related to primary aldosteronism.

Predictor	Crude odds ratio	95% CI	p-value	Adjusted odds ratio	95% CI	p-value
Age >60 years	2.34	1.25–4.38	0.008	2.12	1.01–4.42	0.045
Female	1.54	0.91–2.60	0.091	1.65	1.26–2.17	<0.001
BMI >25	0.63	0.38–1.01	0.060			
Smoking	1.92	1.11–3.32	0.018	2.79	2.51–3.10	<0.001
Duration of hypertension > 6 years	1.40	1.39–1.41	<0.005			
Coronary artery disease	4.22	2.82–6.31	<0.005	2.29	1.57–3.35	<0.001
Obstructive sleep apnea	1.89	0.87–4.08	0.104	1.50	1.07–2.10	0.017
≥ 3 antihypertensive medications	1.29	0.87–1.91	0.189			
Proximal muscle weakness	2.29	0.45–11.52	0.314			
Systolic blood pressure > 160 mmHg	0.96	0.57–1.61	0.880	1.15	1.13–1.16	<0.001
Diastolic blood pressure > 100 mmHg	1.10	0.57–2.14	0.766			
Potassium <3 mEq/L	3.54	1.80–7.04	<0.001	3.72	1.13–12.16	0.030
Sodium >140 mEq/L	1.82	0.64–5.14	0.256			
Bicarbonate >24 mEq/L	1.26	0.42–3.76	0.678			
Fasting blood glucose >126 mg/dL	0.73	0.33–1.61	0.440	0.48	0.31–0.75	0.001
Total cholesterol >200 mg/dL	0.99	0.61–1.61	0.97			
eGFR <60 mL/min/1.73m^2^	2.46	1.17–5.19	0.017	1.79	1.59–2.02	0.001

### Predictive model

**Fig 1:**
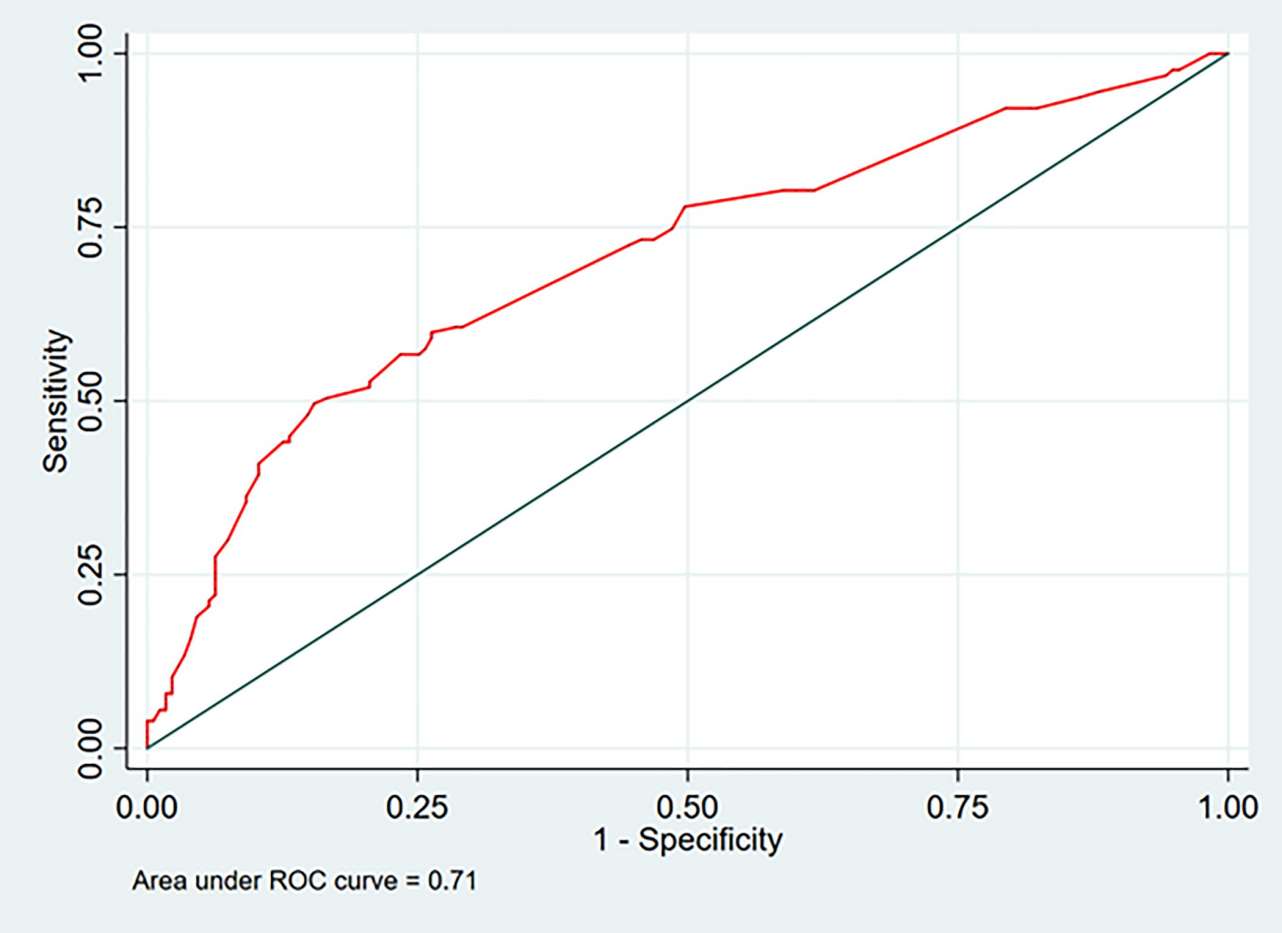
Area under the receiver operating curve (AuROC) of the predictive model for primary aldosteronism incorporating clinical and biochemical factors predicted by the predictive factors (red curved line).

Predictive score was created to predict the probability of patients having PA. The transformed scores ranged from 1.0 to 9.5 with a total score of 42. The scoring system is shown in **Table 3**. The total scores were classified into two groups: a low-risk group (scores 0–7.5) and a high-risk group (scores above 7.5). This cut-off had a sensitivity and a specificity of 72% and 70%, respectively. The predictive ability of the final model represented by AuROC was 0.71 (95% CI: 0.64–0.77) (**Fig 1**).

**Table 3:** Predictive score for the diagnosis of primary aldosteronism.

Predictor	ß-coefficient	Item score	Assigned score
Age >60 years			
• Yes	0.75	5.35	5.5
• No			0
Sex			
• Female	0.47	3.35	3.5
• Male			0
Smoking			
• Yes	0.79	5.64	5.5
• No			0
Coronary artery disease			
• Yes	0.77	5.5	5.5
• No			0
No obstructive sleep apnea			
• Yes	0.37	2.64	2.5
• No			0
Systolic blood pressure > 160 mmHg			
• Yes	0.14	1	1
• No			0
Potassium <3 mEq/L			
• Yes	1.33	9.5	9.5
• No			0
Fasting blood glucose ≤126 mg/dL			
• Yes	0.73	5.21	5
• No			0
eGFR <60 mL/min/1.73m^2^			
• Yes	0.55	3.92	4
• No			0

## Discussion

The key finding in this study is that nine easy-to-obtain clinical and biochemical factors can facilitate the prediction of PA. The predictive performance of the final predictive model which integrated these factors had acceptable predictive performance based on AuROC.

Our study reported a higher prevalence of PA (41.96%) compared to other studies which reported a prevalence of PA ranging from 5–22% [10, 11]. The elevated prevalence of PA in our study could be related to 1) the broader range of indications for PA screening in our study, which included hypertension in young populations and 2) most of the patients having been referred to the endocrine clinic for PA screening, which could indicate that these patients were already seen as having a high likelihood of having PA. The present study identified five additional predictors: female sex, smoking, having coronary artery disease or OSA and low eGFR. Although in a previous study low BMI, a higher number of anti-hypertensive agents and higher serum sodium (>141 mEq/L) showed statistically significant association with PA, in our study, these factors revealed no significant relationship with PA [7].

Previous studies have reported that PA is generally recognized in middle-aged adults in the third to fifth decades of life. This agrees with the finding from our study that the mean age of patients with PA was approximately 47 years old. Nevertheless, recent evidence suggests that PA is becoming more common in the elderly (age over 65 years) [12, 13]. Likewise, our study found that age over 60 years is one of the risk factors related to PA, with a risk 2.12 times than in younger people (aged <40 years). This could result from increased salt sensitivity in the elderly as well as a decrease in PRA from lower production and an increase in PAC from autonomous secretion of aldosterone by aldosterone-producing cell clusters. These factors can lead to relatively high ARR and may result in an increased diagnostic prevalence of PA [13–15]. However, recent evidence suggests that the ARR cut-off for the elderly and extreme elderly should be adjusted to higher values [16], although this recommendation has not yet been endorsed in the most recent guideline.

Regarding the association of gender and PA, the present study showed that being female tended to increase PA risk by 1.65 times compared to males. This finding is similar to other studies which have reported that the prevalence of PA tends to be higher in females than in males. However, in those studies after adjusting for confounders, being female represented no statistically significant increased risk of PA compared to males [7, 17]. Another study reported that female gender is one of the predictors of bilateral adrenal hyperplasia (the predominant subtype identified in our study) [18]. However, there was also evidence that estrogen status or menstrual phase in females can cause a false-positive for direct renin concentration (DRC), but not for PRA, which was the main test in our study [19].

The present study demonstrated that hypokalemia (<3 mEq/L) and moderate to severe hypertension (systolic blood pressure >160 mmHg) are significant predictors for PA, a finding supported by multiple studies. Approximately 30% of patients with PA have hypokalemia [20]. As to the relationship with hypertension, the prevalence of PA was found to increase with the severity of hypertension—8% in moderate hypertension and 13% in severe hypertension [21]. These factors have been included as indications for PA screening in multiple guidelines [5, 8].

Coronary artery disease, OSA and chronic kidney disease (low eGFR) are linked to the pathophysiology of PA by various mechanisms. Increased aldosterone levels in PA patients can activate proinflammatory and oxidative stress and can have profibrotic effects on the vessels contributing to cardiovascular disease [22]. Higher prevalence of OSA in PA patients has been reported to be around 60–70% [23]. The Endocrine Society Guideline also suggests screening for PA in all hypertensives with OSA, suggesting a bidirectional relationship between PA and OSA. Increased aldosterone causes rise in salt and water retention resulting in parapharyngeal edema and worsening of OSA. On the other hand, intermittent hypoxia from chronic OSA leads to increased sympathetic nerve activity and can activate the renin-angiotensin-aldosterone system [24]. In chronic kidney disease, increased aldosterone can damage small and medium vessels and cause nephropathy [25]. Moreover, a study demonstrated that administration of a mineralocorticoid antagonist can improve kidney function and reduce proteinuria in PA patients [26].

Our study revealed that current smokers had a higher risk of having PA. Cigarette smoking could be linked with PA indirectly. Patients with PA who are current smokers may have a higher risk of cardiovascular events caused by increased oxidative stress resulting in endothelial injury [27]. However, a direct association between cigarette smoking and PA has not yet been demonstrated. In fact, a prior study that evaluated risk factors for PA reported that patients’ smoking status had no significant association with PA [28]. The underlying factors related to this issue needed to be explored further.

In a prospective study by German cohort, diabetes and metabolic syndrome were found to be significantly more prevalent in PA than in a matched control population. For that reason, at first we hypothesized that diabetes could be one of the positive predictors of PA. Interestingly, in our cohort fasting study, blood glucose >126 mg/dL had a negative predictive effect on PA (OR 0.48). This unexpected result is similar to that reported by Kietsiriroje et al. [7]. The hypothesis behind our unanticipated finding, is that diabetes can lead to atherosclerosis, thus resulting in a high prevalence of essential hypertension in patients with co-existing diabetes. The prevalence of diabetes in PA patients has been reported to be 17.2% to 23% [29, 30] which is lower than in essential hypertension which has been reported as 16.9–41.9%. This report supported our hypothesis [31].

The present study demonstrated that there is a statistically significant association among easily accessible clinical and biochemical factors and the risk of having PA. The final predictive model combining these factors provided acceptable diagnostic performance. The results help suggest when a clinician should refer a patient for PA screening, especially in institutions where investigative resources are not available. In the future, creation of a predictive risk scoring system would make the system more practical.

The associations between the predictors and PA identified in this study could be explained by underlying physiology and the evidence already presented. To reduce the occurrence of bias, possible confounding factors have been adjusted for in the multivariable model and the criteria for definitive diagnosis of PA as well as other key aspects have been defined. Additionally, cluster analysis was performed to reduce variation in the confirmation tests. A strict criterion for PA diagnosis, i.e., PAC levels >15 ng/dL, was employed to reduce the incidence of false positive diagnoses of PA based on a very low PRA. The relatively large sample size in this study provided sufficient power of analysis.

There are some limitations in this study. First, this is a retrospective and single-center study, a potential source of selection bias which could limit the applicability of the results to other institutions. Second, hypertension in the young population was included as one of the indications for PA screening in our cohort even though this has not been established elsewhere in the standard guidelines. This issue could have had the effect of reducing the incidence of PA identified in our cohort as most of the young (<40 years) patients were diagnosed with essential hypertension. Third, the sample size was not calculated prior to the analysis. However, backward calculation for the power of analysis showed a power of > 0.80 which indicated an adequate sample size to interpret the results. Lastly, some predictors, e.g. young age at onset of hypertension and multiple anti-hypertensive medications used, were not significantly related to PA in our proposed model. These predictors had been reported in multiple studies with the strong associations with PA [32, 33]. Therefore, we recommend that this predictive model should be employed as a supplementary tool to facilitate the diagnosis of PA.

## Conclusions

Numerous clinical and laboratory investigations can facilitate the prediction of PA. This predictive model can be used as an additional tool to allow the physicians in institutions where resources for investigating PA are not readily accessible to decide which patients needed to be referred. Using these predictors could result in time- and cost-savings as the number of PAC, PRA and confirmation tests can be reduced. External validation in future research should be accomplished to confirm the predictive ability of this model.
